# Ground beetle movement is deterred by habitat edges: a mark-release-recapture study on the effectiveness of border crops in an agricultural landscape

**DOI:** 10.1093/jisesa/ieae062

**Published:** 2024-06-17

**Authors:** Magdeline E Anderson, Rachel R Harman, Tania N Kim

**Affiliations:** Department of Entomology, Kansas State University, 123 W. Waters Hall, 1603 Old Claflin Place, Manhattan, KS 66506; Department of Entomology, Kansas State University, 123 W. Waters Hall, 1603 Old Claflin Place, Manhattan, KS 66506; Department of Entomology, Kansas State University, 123 W. Waters Hall, 1603 Old Claflin Place, Manhattan, KS 66506

**Keywords:** biological control, edge effect, dispersal, seed predation, pest management

## Abstract

Border crops can increase beneficial insect biodiversity within agricultural fields by supplementing insects with food and nesting resources. However, the effectiveness of border crops relies on insect movement between adjacent habitats and some insects might consider habitat boundaries as barriers. Therefore, understanding insect movement between habitats is needed to determine the effectiveness of border crops for ecosystem services such as pest control within agricultural habitats. Our objective was to compare ground beetle (Coleoptera: Carabidae) movement across soybean plots that were bordered by corn and grassland habitat to determine whether habitat boundaries were considered barriers of movement to predatory beetles. Using a grid of pitfall traps within these habitats, we conducted a mark, release, and recapture experiment to track and evaluate ground beetle movement patterns. We found that ground beetles stayed in the habitat of their release and that movement between habitats, despite the type of bordering habitat or type of edge, was uncommon. We also found that long-distance movement was rare as most beetles moved less than 5 m (regardless of release or recaptured habitat) and movement was perpendicular to habitat edges. These results suggest that any edge habitat, including agricultural–agricultural boundaries and natural–agricultural boundaries, are likely barriers to ground beetle movement. Therefore, in order for border crops to be effective in pest management by ground beetles, making habitat edges more permeable, especially using techniques such as edge softening, could promote cross-habitat movement and ultimately contribute to natural pest control in agricultural systems.

## Introduction

Beneficial insects provide natural pest control services worth around $4.5 billion USD annually in the United States (Dirzo et al. 2014), but insects have experienced significant declines from agricultural intensification in recent years (Dirzo et al. 2014, Forister et al. 2019, Wagner 2020). Agricultural intensification has led to greater use of and reliance on chemical inputs, which has led to loss in biodiversity, increased prevalence of disease and resistance to chemicals, and depleted local water and nutrient resources (Foley et al. 2005, Meehan et al. 2011, Ickowitz et al. 2019, Raven and Wagner 2021). One way to alleviate beneficial insect decline is through the use of sustainable agricultural practices such as border cropping which provide additional shelter and food resources needed for insect survival and refuges in times of disturbance (Landis et al. 2000, Hopwood et al. 2016, Schulte et al. 2017, Redlich et al. 2018). If movement from the border crops into the adjacent to agricultural fields occurs, then these insects can provide valuable ecosystem services such as natural pest control and pollination services within the managed cropland, ultimately reducing insecticide use and non-target effects on insects and wildlife (Meehan et al. 2011, Rusch et al. 2016, Grab et al. 2018).

The spillover movement of beneficial insects from the border crop into the agricultural field center must be consistent for effective pest control. While mobile generalist insects typically have the physical capability of crossing habitat boundaries and can feed on resources in both habitat types, experimental results on spillover movement are mixed or spatially limited. In a review by Albrecht et al. (2020), the authors found that natural pest control services and pollination decline exponentially with distance from border crop plantings, leaving the interior of fields with little to no pest control. These results suggest that spillover benefits are limited to the edges of the fields. Other studies have found that insects may perceive border crop edges as a movement barrier. For example, Murcia et al. (1995) found shifts in insect abundance and diversity at habitat edges because of the sudden, dramatic change in habitat types, suggesting that habitat edges may limit cross-habitat movement. While many studies have found differences in insect abundance and diversity along distance from edges (as reviewed by Albrecht et al. 2020, Boetzl et al. 2024, Harman and Kim 2024), many studies do not actually measure movement and therefore spillover movement is inferred (Nathan 2008, Joo et al. 2022, Harman and Kim 2024). Because cross-habitat insect movement can be influenced by factors, including edge abruptness (Mader et al. 1990), resource availability (Rand et al. 2006, Knapp et al. 2019), and field management practices (Labruyere et al. 2016), properly documenting insect movement across habitat boundaries are needed to determine the effectiveness of border crops for pest and weed control services.

Ground beetles (Coleoptera: Carabidae) are ideal insects to study habitat use and cross-habitat movement within agricultural systems due to their ability to provide natural weed and pest suppression services (Kromp 1999, Bohan et al. 2011, Kulkarni et al. 2015). However, their effectiveness in seed and pest control is largely dependent on the functional traits of the insect, the local habitat, and the surrounding landscape context. Many ground beetles require permanent natural habitat with suitable moisture levels, vegetation cover, and food availability for survival, overwintering, and oviposition (Wallin 1985, Wallin and Ekbom 1988, Lövei and Sunderland 1996). Often, agricultural field interiors do not provide these necessary conditions, especially as harvesting, tilling, plowing, mowing, and grazing management practices create dangerous seasonal disruptions (Landis et al. 2000, Magura et al. 2017). Thus, to be effective natural pest suppressors, ground beetles must be able to shift between nearby natural habitat and agricultural field centers. The ability to move across habitat boundaries can be linked with functional traits such as dispersal ability, where ground beetles with low dispersal abilities or smaller beetles will have less movement activity than more mobile or larger ground beetle species (Gardiner et al. 2010, Boetzl et al. 2024). Similarly, cross-habitat movement can be affected by functional traits such as diet. In a review by Boetzl et al. (2024), the authors found that the distance functions (activity from the edge of a habitat to the interior) decreased more rapidly with granivores compared with carnivores, likely due to greater seed availability along edges. Finally, cross-habitat movement can depend on the type of habitat. A meta-analysis on edge responses (Magura et al. 2017) found that ground beetles responded differently to edges factors such as level of disturbance (natural or anthropogenic) and management practices (grazing, mowing, or burning) and that most responses were negative to anthropogenic edges. Therefore, the environmental context between the edge of a border crop and agricultural crop could inhibit cross-over between habitats.

Our research objective is to characterize cross-habitat movement of ground beetles between agricultural fields and adjacent habitat types using mark, release, and recapture techniques. Specifically, we asked (i) do ground beetle movement patterns (recapture activity, total distance moved, velocity, directionality) vary among habitat types, (ii) is cross-habitat movement frequent, and (iii) do movement patterns vary with beetle groups? We predicted that movement patterns would vary among habitat types with the slowest movement occurring within the dense grassland interiors compared with corn and soybean which have open understories. We also hypothesized that these effects would vary by beetle size with smaller ground beetles being limited and disproportionately impacted by edges. The results from this study will provide insight into the effectiveness of border crops for conservation practices and area-wide adoption of the practice.

## Methods

### Study Area and Field Site

**Figure 1.**
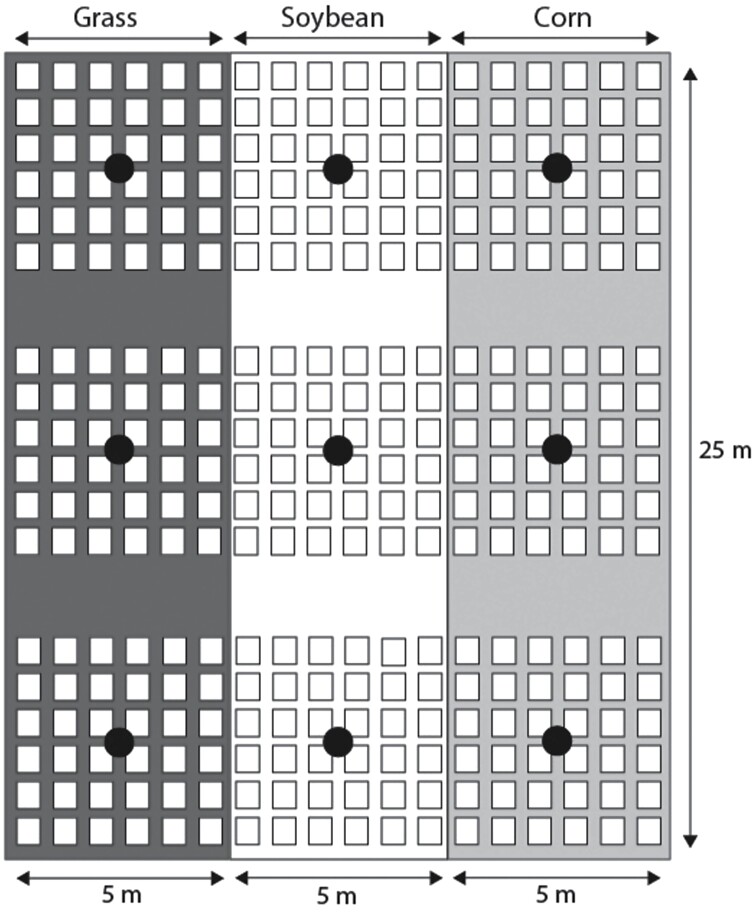
Mark, release, and recapture experimental field site setup. Squares represent pitfall traps, each spaced 1 m apart. Black circles indicate beetle release points at the start of the experiment. The dark grey column represents grassland habitat and the white column represents soybean habitat, and the light grey column represents corn habitat.

To monitor ground beetle movement between different habitat types and across edges, we conducted a mark, release, and recapture experiment at the Agronomy North Farm property of Kansas State University in Manhattan, Kansas, USA (39.212817, -96.601856). The North Farm is a 6.4-hectare agricultural center located on the edge of an urban area. The farm is dominated by row crops such as corn, soybean, wheat, and alfalfa with grassy waterways and strips (grass and forest) scattered throughout the farm for wind protection and erosion and runoff control. The immediate area surrounding the study site included a forested strip (50 m wide × 421 m long) and the study site was historically managed intensively for row crops such as corn, soybean, and wheat. In June, we established strips of soybean and corn (50-m long and 5-m wide) adjacent to a permanent grassy strip that was used for erosion control (Fig. 1). The dense grassy strip was composed of cool and warm season grasses such as Buffalo grass (*Bouteloua dactyloides*) and Tall fescue (*Lolium arundinaceum*) and various forb species including white clover *(Trifolium repens*) and dandelion (*Taraxacum officinale*). The grassy strips was largely left undisturbed throughout the season with infrequent mowing (once or twice per growing season).

For the mark and recapture experiment, a square grid of 30 pitfall traps (hereafter ‘plots’ where plot size = 5 m × 5 m) was placed into each habitat with each trap 1-m apart. A single spatial block consisted of three adjacent plots (90 total traps in total, 5 m × 15 m in size) across the span of the 3 habitats. Each pitfall trap was constructed from a 0.5 liter plastic cup that was punctured with small basal pin holes to allow for water drainage and placed into the ground so that it was flushed with the ground surface (Larsen and Work 2003). A hardware cloth cover (1.3 cm opening; 15 cm × 15 cm square), secured with garden stakes, was used to protect live beetles within each pitfall trap from predators.

There were 3 spatial blocks within the study site, each block spaced 5 m apart (Fig. 1). The spatial blocks were not fenced because we did not want to completely restrict the natural movement patterns of the beetles, and potentially bias our results toward short-distance movement. Thus, the consecutive and continuous positioning of pitfalls within the habitats, plots, and blocks allowed us to assess ground beetle movement across a wide range of distances. However, the uneven topology within the field and disturbances between spatial blocks (e.g., ATV and foot traffic) may have created barriers for cross-block movement. Due to logistical constraints, we did not have all combinations of habitat orders (i.e., corn in the middle or grass in the middle), but we were still able to assess movement across 2 different border types: a soybean–grass border (an example of a semi-natural to agricultural border) and a soybean–corn border (an example of an agricultural–agricultural border).

### Mark, Release, and Recapture Setup

**Figure 2.**
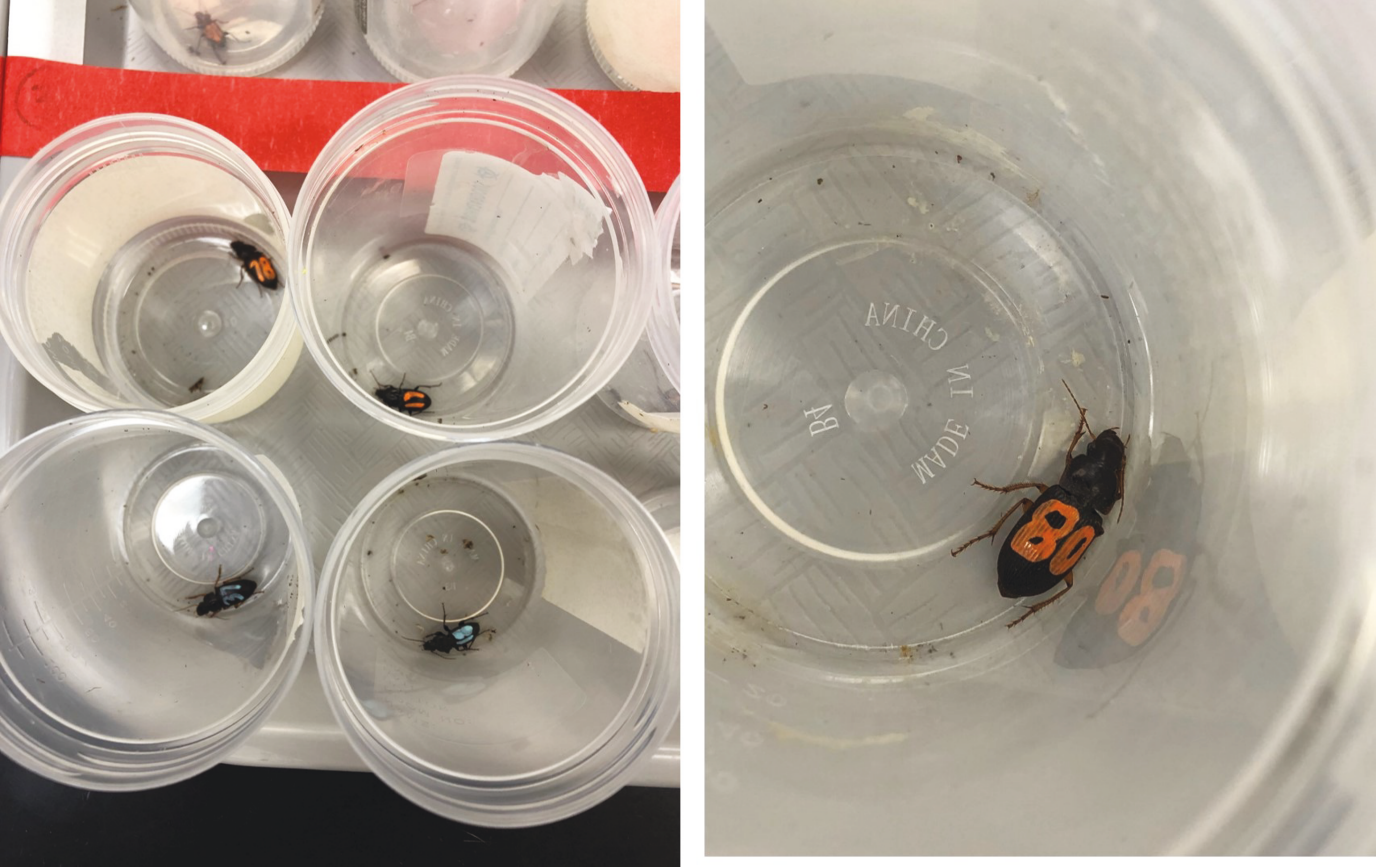
Captured ground beetles are marked with unique identification numbers using paint markers. After 24 h, beetles are released into the study site after beetle identification and body size measurements.

We collected ground beetle specimens from established beetle communities at the North Farm using pitfall traps. Typical mark and recapture studies have low recapture rates (e.g., 13–32% from Elek et al, (2014), 32.9% from Perry et al. (2017)); therefore, to have enough beetles to assess movement patterns, we needed to release a high number of beetles within our experimental site. Because we did not know how many beetles were present at our newly established experimental site and did not want to deplete or disturb these ‘resident’ beetles, we captured beetles off the study site but within the general area in grasslands, corn, and soybean habitat. These initial ground beetle captures (hereafter referred to as ‘original captures’) were brought to the lab and measured for body length. We identified most beetles to the species level, but for some challenging genera (e.g., *Harpalus* and *Pasimachus*), we left identifications at the genus level only because species-level identification would require genitalia dissection. Most of the original captures came from the grass (*n* = 191; 62%), which suggests habitat preference, suitability, and higher abundances within the grassland natural habitat. Beetle captures in grass was over twice the abundance from either the soybean (*n* = 81; 26%) or corn (*n* = 40; 13%) habitats. Captured beetles were kept in separate chambers for up to 24 h with water on a cotton dental wick, and a unique number and color were drawn onto the elytra using nontoxic, low odor, and quick dry TFIVE Oil-based Paint Markers (Fig. 2). Paint markers were chosen for their efficiency and effectiveness for marking insect exoskeletons. We tested this technique in a preliminary greenhouse experiment before transition to the field, and it has also been effectively used in previous ground beetle movement studies (Frampton et al. 1995).

The marked original capture ground beetles were released within the study field after 24 h and re-distributed evenly at 5 release points within each spatial block: (i) grass interior, (ii) grass–soybean edge, (iii) soybean interior, (iv) soybean–corn edge, (v) corn interior (Fig. 1). Beetles released within habitat interiors were placed directly at the center (2.5 m into the 5-m^2^). Beetles released at the edge were placed directly where the 2 habitats met. Thus, we released beetles from 15 different points across the study field. We did not return beetles to their original capture habitats at the original capture densities because grasslands would have received more beetles at the start of the experiment than the other 2 habitats which could have influenced cross-habitat movement potential.

We monitored the traps daily in the mornings until no marked beetles were recaptured (approximately 3 weeks). Results from our preliminary greenhouse study showed that paint started to wear at 2 weeks after marking and became illegible shortly thereafter (T. Kim, unpublished data). We recorded the date, habitat type, plot number, pitfall location, unique ID, and species/genus for each marked beetle event. Then, the marked individual was re-released adjacent to the pitfall trap it was caught in, ultimately making it viable again for another capture. We covered pitfall traps with lids for 6 h after release to prevent beetles from falling immediately back into the traps at the release location. Because ground beetles are largely nocturnal feeders, we removed lids after 6 h (late afternoon). Preliminary greenhouse observations showed that released beetles immediately bury deep below within soil once released therefore accidental falls were unlikely (author, unpublished data). We released a total of 338 individuals representing 10 genera (9 tribes) across 2 sampling sessions in July and August 2022 (Table 1). Marked beetles included *Harpalus* spp. (36%), *Tetracha virginica* (20%), *Pterostichus permundus* (19%), *Scarites vicinus* (11%), *Poecilus chalcites* (9%), *Calosoma affine*(1.8%), *Brachinus alternans* (1.8%), *Chlaenius tomentosus* (1.2%), *Cicindela punctulata* (0.3%), and *Pachimachus* spp. (0.3%). For simplicity, we refer to the beetles by their genera because we captured only 1 species per genera (with the exception of *Harpalus* and *Pachimachus*).

**Table 1.** Number of beetles released and recaptured within experimental plots sorted by tribe and species.

Tribe	Species	Released		Recaptured	
		Number	Percent	Number	Percent
Brachinini	*Brachinus alternans*	6	1.78	1	1.43
Carabini	*Calosoma affine*	6	1.78	2	2.86
Chaeleniini	*Chlaenius tomentosus*	4	1.18	–	–
Cicindelini	*Cicindela punctulata*	1	0.30	–	–
Harpalini	*Harpalus spp.*	122	36.09	20	28.57
Megacephalini	*Tetracha virginica*	68	20.12	28	40.00
Pasimachini	*Pasimachus spp.*	1	0.30	–	–
Pterostichini	*Pterostichus permundus*	65	19.23	11	15.71
Pterostichini	*Poecilus chalcites*	29	8.58	3	4.29
Scaritini	*Scarites vicinus*	36	10.65	5	7.14
Total		338	100%	70	100%

### Data Analysis

We calculated the total distance moved and distance traveled per day for each captured ground beetle based on release and recapture locations. We constructed a dispersal kernel to determine the distribution of movement distances for all recaptured beetles. We also compared recapture activity (number of recaptures, Table 1) by habitat type. Because of the spatial locations of the habitats, soybean (Fig. 1; middle column) had a greater likelihood of recaptures than the neighboring grassland and corn habitats, we did not compare the total number of captures within each habitat as this would bias recaptures in favor of soybean. Therefore, we standardized the number of recaptures based on the number of released within a fixed distance from the capture location using the dispersal kernel. Specifically, the recapture activities were calculated as the number of recaptures/the number released within 5 m radius (typical distance moved based on the dispersal kernel—see Results section). Because soybean was at the center of the experimental site, the number released within 5 m was higher than those released corn and soybean. For recaptured beetles (beetles with more than 2 movement steps), we also compared movement directionality (angle of movement relative to the release location) to assess whether the trajectory of the beetle movement changed with the previous recapture. We calculated the Pearson correlation coefficient between the angle of the movement at one timestep against the movement at the next time step to determine whether there was a correlation between the two angles (Harman 2020). Using these data, we also compared whether movement was away or toward habitat edges to determine whether beetles were avoiding edges. We compared standardized recapture activity, distance traveled per day (hereafter velocity), and total distance moved using analysis of variance (ANOVA) and predetermined Tukey post hoc multiple comparison of means. Chi-square contingency tests were used to assess the distributions and compare frequencies between ground beetles released and recaptured in different habitat locations. All statistical analyses were conducted in R (v 4.3.2; R Development Core Team 2020)

## Results

A total of 70 marked ground beetles representing 7 genera were recaptured 120 times for a 37.85% recapture rate (Table 1). Of the recaptured genera, *Tetracha* was recaptured the most (*n* = 28) and the highest rate (40%), while *Brachinus* was the fewest (*n* = 1 or 1.4%). Three of the released genera, *Chlaenius*, *Cicindela*, and *Pasimachus*, were not recovered. These were the least common genera from the original captures, as only 6 individuals across the 3 genera were released.

**Figure 3.**
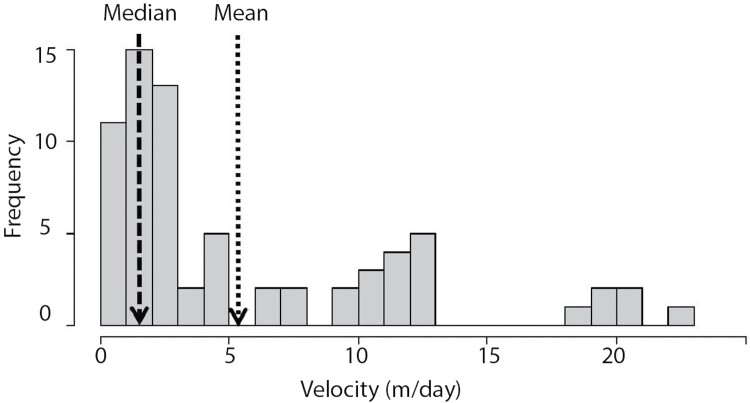
Histogram of ground beetle velocity (meters/day). The dashed line signifies the median velocity (2 m/day) and the dotted line signifies mean velocity (5.39 m/day).

**Figure 4.**
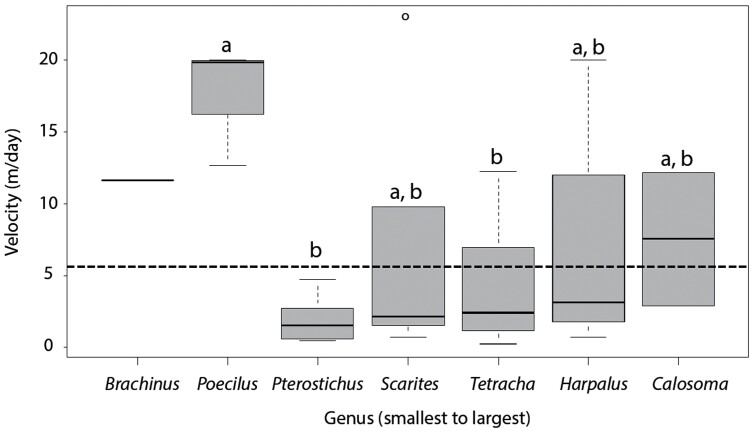
Genus-level differences in velocity (m/day). The dashed line signifies the overall average velocity (5.72 m/day). *Brachinus* was not included in the statistical analyses because only 1 individual was recaptured. Letters denote statistical significance in velocity among genera.

Overall, the majority of the beetles remained close to their release point (Fig. 3). The mean velocity (travel distance per day) was 5.72 m/d (median = 2.8 m/day) and over half the ground beetles (*n* = 46; 58 % of recaptures) moved between 0.5 and 5 m/day. Very few beetles (*n* = 3; 4.29 % of recaptures) moved over 20 m/day, with the maximum being 22.85 m/day. For beetles captured multiple times (35.7% of recapture), the average number of recaptures was 1.7 times (min = 1 time; max = 7 times, median = 1 time) and the averaged total distance traveled was 15.1 m (min = 0.5 m and 48.8 m), with 25% of these beetles moving<5 m in total. The average velocity varied by genera (Fig. 4: *F* = 4.84, df = 5, 63, *P* < 0.001). The two smallest beetle groups (*Brachinus* and *Poecilus*) had the greatest velocity (*Poecilus* moving the farthest at 17 m/day (range = 12–20 m/day) whereas third smallest genus (*Pterostichus)* had the lowest velocity (mean = 2.0 m/day, range = 0.5–4.8 m/day), which is lower than the overall average (2.0 m/day). A few individuals from the *Scarites* and *Tetracha* genera had the highest velocity (20–22 m per day) and total distance traveled (20–46 m in total). However, the total distance moved throughout the experiment did not vary by genera (*F* = 1.81, df = 5,63, *P* = 0.122).

**Figure 5.**
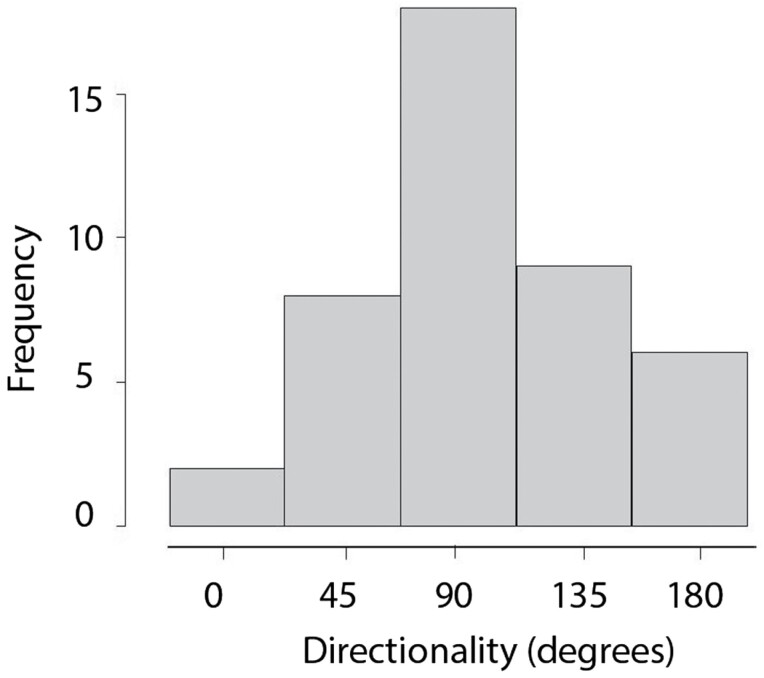
Histogram of the directionality of movement (in degrees). 0 and 180°C indicate that movement direction is parallel to edges while 90°C is perpendicular.

The total distance moved and velocity did not vary with release habitat (total distance moved: *F* = 0.601, df = 4, *P* = 0.663; velocity: *F* = 0.369, df = 4, *P* = 0.83) or recapture habitat (total distance moved: *F* = 0.294, df = 4, *P* = 0.746; velocity: *F* = 0.685, df = 4, *P* = 0.506). For beetles captured more than once, the directionality of movement was uncorrelated with the previous movement direction (*r* = -0.016, *t* = −0.115, df = 48, *P* = 0.90). In general, directionality was perpendicular to the edges suggesting that beetles moved away from or toward the edges (*χ*^2^ = 34.248, df = 4, *P* < 0.001, Fig. 5). This pattern was consistent regardless of recapture and release habitat types.

Recapture rates (number of recaptures/releases within 5-m radius) were similar between the grass (2.6 recaptures/release) and soybean (2.7 recaptures/release) habitats, but lower in the corn (1.8 recaptures/release) habitat. Ground beetles largely remained within the same habitat that they were released in (Table 2; *χ*^2^ = 22.73, df = 4, *P* < 0.001, *n* = 97). On average, 72.5% of recaptures stayed in the same habitat that beetles were released in while 25.5% moved to a different habitat. When beetles were released directly on the edge of two habitats, there was no preference for one habitat over the other (Table 3; *χ*^2^ = 3.6, df = 2, *P* = 0.162, *n* = 37). One exception was *Harpalus* which, when released in the centers of either grass or corn, were frequently recaptured in soybean. Furthermore, when *Harpalus* was released along habitat edges (natural–agricultural and agricultural–agricultural), 100% of all *Harpalus* recaptures were in soybean, indicating strong preference for soybean. No other genera displayed a preference for habitat type, suggesting that these ground beetles are well adapted to different habitat types.

**Table 2.** Contingency table showing percent of beetles recaptured relative to their initial release location. Most beetles remained in the same environment they were released in (highest percentages on the diagonal, mean = 72.5% of recaptures). Column headings represent released habitats whereas row headings represent recaptured habitat. Totals (column or rows) do not equal 100% because some beetles were captured multiple times and not all beetles were recaptured.

Recaptured habitat	Released habitat		
	Grass	Soy	Corn
Grass	69.40%	17.10%	4.50%
Soy	16.70%	75.60%	22.70%
Corn	8.30%	7.30%	72.70%

**Table 3.** Contingency table showing percent of ground beetles captured in soy, grass, and corn after released from a grass-soy edge or a corn-soy edge.

Recaptured habitat	Released edge location	
	Grass-Soy Edge	Corn-Soy Edge
Grass	33.30%	9.10%
Soy	41.20%	35.50%
Corn	8.30%	36.40%

Movement across habitat boundaries was uncommon (26% of captures). Of the recaptures across habitats, 69% of theses recaptures traveled across the grass-soybean edge (*n* = 15) and 31% resulted in a soybean–corn edge crossing (*n* = 14). There was no preference in the directionality across these natural–agricultural border crossings (*n* = 7 events from grass to soybean, *n* = 8 from soybean to grass). Similar patterns were found for the agricultural–agricultural border (*n* = 7 events from soybean to corn, *n* = 7 from corn to soybean). The edge crossing events were dominated by three genera, *Tetracha* (38%), *Harpalus* (27%), and *Poecilus* (17%). We documented at least one crossing event for all recaptured genera.

## Discussion

Results from our mark, release, and recapture study offer a better understanding of cross-habitat spillover of beetles in human modified landscapes and deliver insight on the effectiveness of border cropping practice for both grower management and ecosystem functioning. Our results show that ground beetles were not very active as they stayed in their release environment. Despite an original preference for grassland, ground beetles in our study did not have strong preference for habitat type, with the exception of one genus *(Harpalus)*. These findings suggest that generalist ground beetles are adapted to survive in a variety of habitats, which could be beneficial for use as a biological control agent for numerous pest insects. Ground beetle movement was largely perpendicular to the edges, indicating that movement was not random and that beetles either avoided or moved toward edges. Since cross-habitat movement was uncommon, no matter the edge type, ground beetles are likely avoiding edges as they might perceive the edges as barriers to movement. Therefore, for border crop practices to be successful for natural pest suppression, the implementation of techniques to facilitate and promote cross-habitat movement of these generalist beetles are needed.

Even though our spatial blocks were not fenced in, we found that ground beetles moved short distances (average = 5.72 m/day), and velocity did not vary with habitat type despite our initial hypothesis of slower movement in dense vegetation. However, these distances match previous studies of ground beetles in other open habitat systems (Brouwers and Newton 2009) and meta-analyses (Allema et al. 2015), where ground beetle movement was on average 2.05 m—9.2 m per day. Our dispersal kernel was positively skewed where a greater proportion of captured beetles moved short distances. Even though long-distance movement (20–22 m/day) was in the minority, these long-distance dispersers are important for maintaining metapopulation structure, particularly within highly fragmented agricultural systems (de Vries et al. 1996, Petrovskii et al. 2014, Bailey et al. 2021). While short-distance movement was common, our tracking method may have limited the actual distance traveled (Brouwers and Newton 2009). For example, in a review by Brouwers and Newton (2009), harmonic radar captures longer distance movement whereas mark-recapture techniques generally capture shorter distance movement, suggesting that we may have been conservative in actual movement observed. Our recapture rates were 37.85% so it is possible that the actual distance moved is higher as the uncaptured beetles could have traveled farther distances than our experimental set up could assess. Furthermore, because movement is often times non-linear, our displacement estimates may also underestimate movement distances, as most ground beetles implement random search tactics and will turn frequently while searching for food or optimal conditions (Lövei and Sunderland 1996).

Most of the ground beetles used for the experiment were originally captured in nearby grasslands which suggests habitat preference for grasslands. It is well documented that natural habitats are higher in ground beetle diversity and abundance compared to crop habitats, likely due to fewer disturbances and more food resources (Rischen et al. 2021). However, once marked beetles were released into the experimental field, they remained close to their release point, no matter the location or habitat. This suggests that ground beetles in our system are well adapted to different environments. The ground beetles’ continued presence within soybean and corn interiors could signal that they are benefitting from agricultural systems, especially because ground beetles are opportunistic feeders (Erwin 1979). For *Harpalus*, there was a preference for soybean, suggesting that soybean habitat contains different food sources compared with grassland. Many ground beetles, particularly *Harpalus*, are omnivorous and known to eat a diversity of weed seeds in addition to agricultural pest insects (Lövei and Sunderland 1996, Bohan et al. 2011, Kulkarni et al. 2015). It is possible that on the short term, the three habitats studied provided adequate amounts of food resources, but grasslands provide a permanent and undisturbed nesting habitat during the rest of the year. Other studies have found that beneficial insects, including ground beetles, use agricultural habitats when conditions are favorable, such as at peak growing season, but then retreat to natural areas when conditions are hostile, such as post crop-harvest (Wissinger 1997). Therefore, providing a stable, less disturbed refuges adjacent to croplands would be necessary to promote ground beetle abundance and diversity.

Although ground beetles were found to utilize all three habitats and would benefit from cross-habitat movement, our results show that when confronted with habitat edges, that ground beetles tended to avoid cross-habitat movement. The limited cross-habitat movement and avoidance of edges in movement directionality suggest that edges might be perceived as movement barriers to ground beetles. While our experimental design did not include all combinations of the three habitats in different orders, our movement results with soybean as the interior habitat fit with previous studies that have also shown that many edge types, including dirt roads, paved roads, and grassy banks, all significantly reduce ground beetle movement (Duelli et al. 1990, Mader et al. 1990, Frampton et al. 1995). However, previous studies have found that different edge types are more permeable than others. Specifically, ground beetle movement across agricultural–agricultural edges were greater than natural–agricultural edges due to less variability in ground cover (Duelli et al. 1990, Allema et al. 2015). While soybean and corn had similarly less ground cover than grassland in our experimental system, we did not find any differences in movement with edge types (corn to soy, and grass to soy). Habitat differences in other abiotic factors, such as humidity and canopy cover, could influence cross-habitat movement therefore to promote cross-habitat movement, adjacent habitats would need to be similar in structure, or edges need to be softened with the use of transitional areas in between habitat types.

We found movement patterns to differ among beetle genera. First, we predicted that larger beetles would move greater distances than smaller beetles (Terlau et al. 2023); however, we found that recapture activity and distance moved did not correlate with body size, even though body sizes varied 2-fold (11.6 mm for *Brachinus* to 28 mm for *Calosoma*). One possibility could be that our range of sizes were not large enough to detect size-related differences in movement; however, work by Terlau et al. (2023) examined ground beetles across a similar range (8–21 mm) and found a positive relationship between beetle size and speed. Instead, we found that the velocity was genera specific where *Poecilus* had the greatest velocity compared with the other beetle genera (average 17.5 m/day) and *Pterostichus* had the lowest velocity (average 2 m/day). Both genera are considered smaller beetles; however, they do vary from the other genera in other aspects. Specifically, *Poecilus* differs because it is predominantly diurnal feeding (Lövei and Sunderland 1996) while *Pterostichus permundus* is primarily a forest-dwelling species (Brouwers and Newton 2009). Insect foraging and movement is generally linked to abiotic conditions such as light and temperature (Hannigan et al. 2022, Terlau et al. 2023), prey availability (Bailey et al. 2021), and predator avoidance (Allema et al. 2015). In the case of the diurnal *Poecilus*, greater velocity could be in response to warmer daytime temperatures while lower velocity of the forest-dwelling *Pterostichus* could be a response to avoiding predators in open agricultural fields. Second, we found that cross-habitat movement only occurred with three ground beetle genera. A meta-analysis conducted on ground beetle abundances and assemblages at natural and anthropogenic forest edges showed that edge response differed among species (Magura et al. 2017). In our study, *Tetracha* accounted for greater than one-third of the total crossing events. These big-headed tiger beetles are large, nocturnal, flightless predators that rely on their eyesight to capture prey and are common around lake or river edges (Ball and Bosquet 2010). Their size, affinity for edges, and aggressive predator tendencies likely contribute to their agricultural edge crossing abilities. In contrast, we only recorded a single crossing event of *Brachinus* between the soybean and corn fields. This bombardier beetle’s smaller size, in comparison to the big-headed tiger beetle, probably made crossing an edge more difficult. It has been reported that smaller beetles, with less dispersal ability, are likely more affected by field boundaries (Frampton et al. 1995). Understanding how these life-history traits affect beetle movement can offer insight into why spillover occurs for some insect groups and not others.

### Implications for the Border Cropping Practice

Overall, our findings show that ground beetles are habitat generalists and are active in all three habitat types. Additionally, the infrequent habitat crossings show that edges are barriers to movement. In this way, for the border cropping practice to have benefits for pest suppression, further management might be needed to promote cross-habitat movement. Specifically, the implementation of various techniques, such as softening edges or using lures, could make habitat edges more permeable and could promote cross-habitat movement further infield. A softer, more gradual, and smooth transition between habitat types would facilitate cross-habitat movement. Magura et al. (2017) found that highly managed anthropogenic edges were much more impenetrable in comparison to natural edges. Because our ground beetles displayed few edge crossings, this practice could potentially impact movement and natural pest control. We also found that movement frequency, habitat preferences, and cross-habitat movement did vary with beetle genera. Specifically, *Poecilius* (a diurnal genera) moved greater distances per day, *Harpalus* (an omnivorous genus) preferred soybean habitat, and only two genera (*Harpalus* and *Tetracha*) crossed habitat edges. Therefore, in order to promote spillover from border crops into croplands, management efforts targeting specific ground beetle species (or genera) might be needed to increase the effectiveness of border crops for large-scale agricultural pest and weed suppression.
